# Epigenetic Mechanisms Influencing Therapeutic Response in Breast Cancer

**DOI:** 10.3389/fonc.2022.924808

**Published:** 2022-06-14

**Authors:** Amaia Arruabarrena-Aristorena, Eneda Toska

**Affiliations:** ^1^ Center for Cooperative Research in Biosciences (CIC bioGUNE), Basque Research and Technology Alliance (BRTA), Bizkaia Technology Park, Derio, Spain; ^2^ Ikerbasque, Basque Foundation for Science, Bilbao, Spain; ^3^ Traslational Prostate Cancer Research Lab, CIC bioGUNE-Basurto, Biocruces Bizkaia Health Research Institute, Derio, Spain; ^4^ Department of Oncology, Sidney Kimmel Cancer Center, Johns Hopkins University School of Medicine, Baltimore, MD, United States; ^5^ Department of Biochemistry and Molecular Biology, Johns Hopkins School of Public Health, Baltimore, MD, United States

**Keywords:** breast cancer, epigenetics, estrogen receptor - ESR1, endocrine therapy, transcription factor, chromatin regulation

## Abstract

The majority of breast cancers are estrogen receptor (ER)+ and agents targeting the ER signaling pathway have markedly increased survival for women with breast cancer for decades. However, therapeutic resistance eventually emerges, especially in the metastatic setting. In the past decade disrupted epigenetic regulatory processes have emerged as major contributors to carcinogenesis in many cancer types. Aberrations in chromatin modifiers and transcription factors have also been recognized as mediators of breast cancer development and therapeutic outcome, and new epigenetic-based therapies in combination with targeted therapies have been proposed. Here we will discuss recent progress in our understanding of the chromatin-based mechanisms of breast tumorigenesis, how these mechanisms affect therapeutic response to standard of care treatment, and discuss new strategies towards therapeutic intervention to overcome resistance.

## Introduction

Over 250,000 breast cancer cases are diagnosed in the US each year (1). The majority of breast cancers (70%) express estrogen receptor (ER) and are treated with agents targeting the ER signaling pathway (2). Endocrine therapy has markedly improved the lives of breast cancer patients for decades. More recently the addition of PI3K inhibitors (alpelisib) or CDK4/6 inhibitors (palbociclib, ribociclib, abemaciclib) to antiestrogens has significantly prolonged progression-free survival (PFS) in comparison to anti-estrogens alone in patients with ER+ metastatic breast cancer (3–6). However, *de novo* and acquired resistance to these treatments remains a major challenge and a high research and clinical priority (5, 7). Research over the past decade has unmasked a key contribution of disrupted chromatin and transcriptional regulatory processes to cancer and in particular, ER+ breast cancer. Laboratory-based functional genetic screens (siRNA, CRISPR), together with molecular profiling of biopsies from patients resistant to targeted therapies have unveiled chromatin modifiers and transcription factors linked to metastatic resistant tumors. Here we review several key epigenetic mechanisms dictating breast cancer tumorigenesis and their roles in therapeutic response in ER+ breast cancer. We also discuss the role of epigenetic factors as promising new targets for overcoming therapeutic resistance in breast cancer.

## Epigenetic Mechanisms of ER Signaling

**Figure 1:**
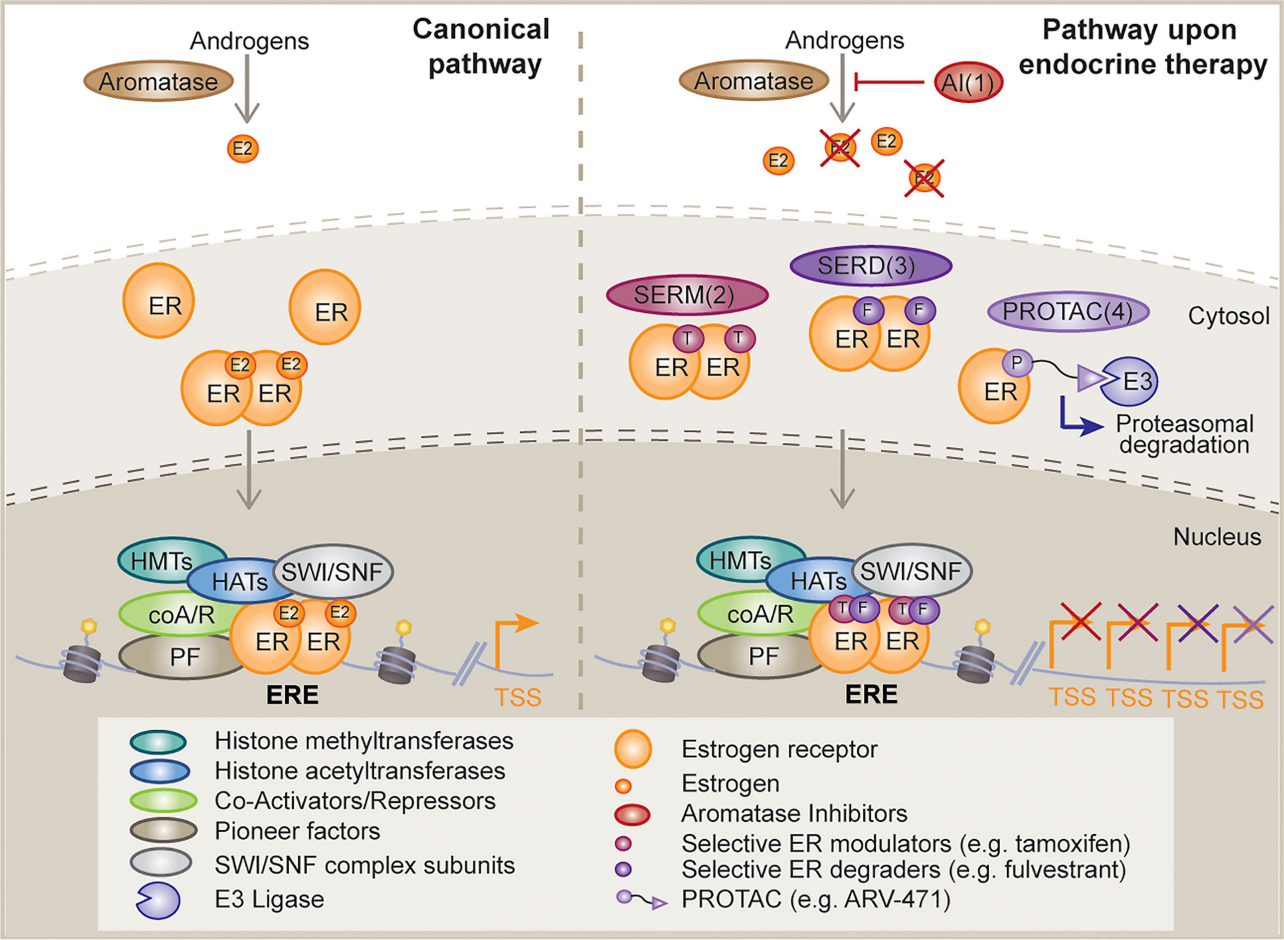
Mechanism of action of classical and novel endocrine therapies. Schematic diagram depicting, on the left, the canonical estrogen receptor (ER) activating signaling cascade and potential downstream interactor types; and on the right, the different mechanisms of disruption of this signaling pathway upon distinct endocrine therapy strategies, such as, aromatase inhibitors (AI, 1), selective ER modulators (SERMs, 2), selective ER degraders (SERDs, 3) and proteolysis targeting chimeras (PROTACs, 4). E2, estrogen; ER, estrogen receptor; T, tamoxifen; F, fulvestrant; E3, E3 ligase; HMT, histone methyltransferase; HAT, histone acetyltransferase; coA/R, co-activator/repressor; PF, pioneer factor; TSS, transcription start site; ERE, estrogen response element.

ER is a member of the endocrine or steroid receptor subfamily of nuclear receptors, also known as Type I nuclear receptors. As members of this subfamily, ER and other nuclear receptors such as AR, share a ligand binding-driven activation mechanism, meaning they bind chromatin upon steroid stimulation. Moreover, they have a common structural domain distribution that ensures the presence of: a variable binding site for interaction with cooperating factors at the N-terminus, namely activation function 1 (AF1); a DNA binding domain (DBD); the interdomain hinge, which encompasses a nuclear localization sequence (Hinge); and a specific ligand-binding domain, which also enables the interaction with additional cofactors and is known as activation function 2 (LBD, AF2) (2). While these nuclear receptors exist at the plasma membrane (in their monomeric form) and in the nucleus (dimerized), the major pool of ER (85%) localizes to the nucleus upon estrogen stimulation. Upon the hormonal trigger ER monomers change conformation and dimerize (**Figure 1**). Dimerized ER is then translocated into the nucleus where it specifically binds at estrogen responsive elements (EREs), with the subsequent induction of the estrogen response (8). This leaves approximately 5% of ER, in its monomeric conformation, that travels to the cell surface upon palmitoylation and subsequently associates with Caveolin-1. ER relies on interactions with several co-regulator proteins to promote or inhibit its activity (**Figure 1**). Examples include the p160 family proteins (SRC1, GRIP1 and AIB1), namely co-activators, as well as nCOR1 and SMRT, which function as repressors Other cooperating factors consist of pioneer factors, such as FOXA1 (9) or GATA3 (10); ATP-dependent chromatin remodelers, for instance SWI/SNIF complex subunits BRG1, BRM or BAF57; and finally, histone and DNA modifiers, such as acetyltransferases (HATs), deacetylases (HDACs), methyltransferases and demethylases. p160 proteins are responsible for recruiting the co-activators p300 and CBP, and histone methyltransferases CARM1 and PRMT1. p300 and CBP have intrinsic and specific histone acetyltransferase (HAT) activity for H3K14, H4K5, H4K8 and additional lysine residues in histone 2A and 2B subunits. ER can also indirectly interact with other HATs. An example is p300/CBP-associated factor (PCAF), which can self-acetylate or be acetylated by p300, while it acetylates H3K9 and H3K14. On the other hand, PRMT1 is responsible for H4R3 methylation, while CARM1 methylates H3R2, H3R17 and H3R26. However, these methylation modifications are reversible through the action of the lysine-specific demethylase 1 (LSD1), which can specifically demethylate H3K4 and H3K9 (2). Bromodomain protein BRD4 has also been shown to be required for ER-dependent enhancer activation and transcription (11). Finally, ER also interacts with other epigenetic regulators, such as Polycomb repressive complex 1 (PRC1) (12). RING1B, a core PRC1 subunit and a histone H2A ubiquitin ligase, is overexpressed in luminal breast cancers and is a crucial regulator of the dynamic, liganded-ER transcriptional programs (13).

## Genomic Alterations and Standard of Care Targeted Therapies in ER+ Breast Cancer

Breast cancer was first demonstrated to be a hormone-driven disease by George Beatson in 1896 (14), long before the discovery of ER by Elwood Jensen and Jack Gorski in 1967 (15, 16). These findings ignited the development of endocrine therapies and personalized medicine. Currently, the ER signaling pathway is targeted by selective ER modulators (SERMs) (e.g., tamoxifen), which compete with estrogen for binding to ER; selective ER degraders (SERDs) (e.g., fulvestrant) that are thought to induce ER protein degradation or block ER activity; and aromatase inhibitors (AIs) (e.g., anastrozole, letrozole, exemestane), which deplete estrogen sources by inhibiting the conversion of androgens to estrogens (5) (**Figure 1**). Of note, it has been recently shown that a number of fulvestrant-like ER degraders suppress ER dependent-transcription mainly by slowing the intra-nuclear mobility of ER (17). In addition, a number of next generation oral SERDs with potentially better pharmacological properties than fulvestrant are in clinical trials (18) (**Figure 1**). These include rintodestrant (phase I, NCT03455270), elacestrant/RAD1901 (phase 3, NCT03778931), giredestrant/GDC-9545 (phase II, NCT04436744), amcenestrant/SAR439859 (phase III, NCT04478266), camizestrant/AZD-9833 (phase III, NCT04711252), and LY3484356 (phase I, NCT04188548) among others. Recent press news has revealed that giredestrant and amcenestrant did not meet their primary endpoint of improving progression free survival (PFS) while the EMERALD trials of elecastrant showed a 30% reduction in PFS during the 2021 San Antonio Breast Cancer Symposium. Novel therapies that are also in the clinic include the SERMs lasofoxifene (phase II, NCT03781063), bazedoxifene (phase I/2, NCT02448771), the proteolysis-targeting chimeras (PROTAC) ARV-471 (phase 1/2, NCT04072952), and the selective estrogen receptor covalent antagonist (SERCA) H3B-5942 (phase I, NCT04288089). Preclinical work has shown significant single-agent antitumor activity of H3B-5942 in wild-type ER and mutant ER xenograft models that was superior to fulvestrant and whose potency could be improved further in combination with CDK4/6 or mTOR inhibitors (19). The development of these new bioavailable drugs against ER raises hopes that they may improve the lives of patients with resistant ER+ breast cancer.

One of the hallmarks of ER+ breast cancer is its dependence on the phosphatidylinositol-3-kinase (PI3K) pathway, which is highlighted by the frequency of activating mutations in the gene *PIK3CA* (~40%), coding for the catalytic subunit of PI3K. Other alterations that can lead to hyperactivation of the PI3K pathway in breast cancer include *ERBB2* and *AKT* mutations, and deletions, nonsense and loss-of-function missense mutations in the tumor suppressor PTEN (20, 21). Aberrant activation of the PI3K pathway promotes acquired resistance to anti-ER therapies in preclinical models (22, 23). The clinical significance of the PI3K pathway in ER+ breast cancer has been shown by the approval of PI3K pathway inhibitors in this setting. The mTORC1 inhibitor everolimus, which inhibits a critical PI3K pathway node was approved first in combination with AIs in metastatic breast cancer patients that are refractory to endocrine therapy (24, 25). More recently, in patients with metastatic ER+/*PIK3CA* mutant breast cancer, the addition of the PI3Kα inhibitor alpelisib was approved in combination with fulvestrant (4). The AKT inhibitor capivasertib in combination with fulvestrant has also shown benefit in preliminary studies in endocrine refractory ER+ breast cancer. This combination may be effective in *AKT* or *PTEN* mutant breast cancer (26).More recently, it has been shown that proline rich 11 (PRR11) overexpression amplifies PI3K signaling and promotes endocrine therapy resistance in breast cancer, suggesting that the ER+/PRR11-amplified breast cancers subgroup of tumors can also benefit from treatment with PI3K inhibitors and antiestrogens (27).

While ER and PI3K pathway alterations are the most frequent oncogenic drivers in ER+ breast cancers, other drivers such as cyclin D1 are expressed at a high level, with or without gene amplification. ER activates the CCND1 promoter, while cyclin D1 also binds to and facilitates ER transcriptional activity, reflecting the possible dependence of ER+ tumors on cyclin D1 to initiate the G1-to S-phase transition. Accordingly, addition of CDK4/6 inhibitors (e.g., palbociclib, ribociclib, abemaciclib) to anti-ER therapy have markedly prolonged survival compared to anti-ER therapy alone in ER+ metastatic breast cancers (3, 6). Thus, after decades of endocrine therapy as a single agent the approval of everolimus, alpelisib, and CDK4/6 inhibitors has led to significant progress in breast cancer management. ERBB2 amplification/HER2 overexpression is also found in 10% of ER+ breast cancers and the current standard of care for ER+/HER2+ is a combination of anti-ER and HER2 inhibitors (28). Rare HER2 mutants found in 5% of endocrine-resistance metastatic breast cancer have also been associated with endocrine resistance (29). However, the combination of the HER2 inhibitor neratinib with fulvestrant has shown promise in this setting (30).

An enrichment in mutations in genes coding for transcription factors (TFs), such as GATA3, CTCF, FOXA1, and MYC (31); and chromatin modifiers, such as the histone methyltransferases (KMT2B, KMT2D, KMT2E) and histone demethylases (KDM4A, KDM5B, KDM5C, KDM6A) (20), and SWI/SNF complex subunits (ARID1A, ARID2) (31), have also been observed in ER+ breast cancer. However, the functional relevance of most of these alterations remain to be identified. More recently, the Breast International Group (BIG) molecular screening initiative AURORA identified a driving role for somatic mutations in the TF *GATA1* and the chromatin regulator *MEN1* among 381 breast cancer patients (32). Apart from *TP53*, *PIK3CA*, *ESR1*, and *GATA3*, the most frequent alterations in primary and/or metastases in the AURORA cohort were found in the lysine histone acetyltransferase KAT6A (32). KAT6A is also amplified as part of the 8p11 amplicon in 10-15% of breast cancers. In addition to the aforementioned alterations, breast cancers also harbor a variety of rare mutations with low prevalence across subtypes, highlighting the heterogeneity of breast cancer and the need to study these variants to develop targeted therapies matched to the specific molecular alteration of each patient’s tumor.

## Transcription Factors and Chromatin Modifiers Affecting Therapeutic Outcome in ER+ Breast Cancer

In the contemporary era, next-generation sequencing technologies, such as whole genome sequencing (WGS) and whole-exome sequencing (WES), have expanded the landscape of genomic variations occurring in cancer, particularly in hormone-dependent breast cancer. Among the most frequently altered genes we find a variety of transcription factors and chromatin remodelers (31, 33). We will focus this part of the review on those examples proven to directly or indirectly impact patient response to standard of care treatment. For more detailed review of chromatin-based mechanisms in breast cancer see Morey and colleagues (7).

### Alterations in Transcription Factors Affecting Endocrine Therapy Response

Despite the initial success of targeted endocrine therapies to tackle ER-driven programs, resistance to such treatments eventually emerges. Mutations in ER itself are a prominent example of driver alterations. Recurrent *ESR1* mutations localized at the ligand-binding domain (e.g. mutations at residues T537 and D538) have been shown to confer ligand-independent activity, establishing a range of sensitivity to the distinct ER antagonists and hormone depleting agents, such as AIs (34–36). Besides promoting a constitutively active agonist conformation, these alterations lead to an altered ER cistrome and the induction of a pro-metastatic transcriptome (37). ER relies on multiple cooperating factors, such as pioneer factors and coregulators, to regulate the estrogen response. FOXA1 is a driver of luminal breast cancer identity (38), and a crucial pioneer and cooperating factor for nuclear receptor activity (9, 39, 40). Recent work aimed at elucidating the mechanisms that regulate FOXA1 binding to the chromatin, has identified the lysine-specific demethylase 1A (LSD1) to positively regulate FOXA1 binding by demethylating lysine 270 on FOXA1 (41). LSD1 inhibition affected androgen response in prostate cancer and sensitized tumors further to anti-AR therapy (41). We have also shown that FOXA1 binding profiles are influenced by the SWI/SNF complex (42) and the histone methyltransferase KMT2D in breast cancer (43), suggesting a possible role for the chromatin landscape to evoke further differences in DNA binding for FOXA1.

**Figure 2:**
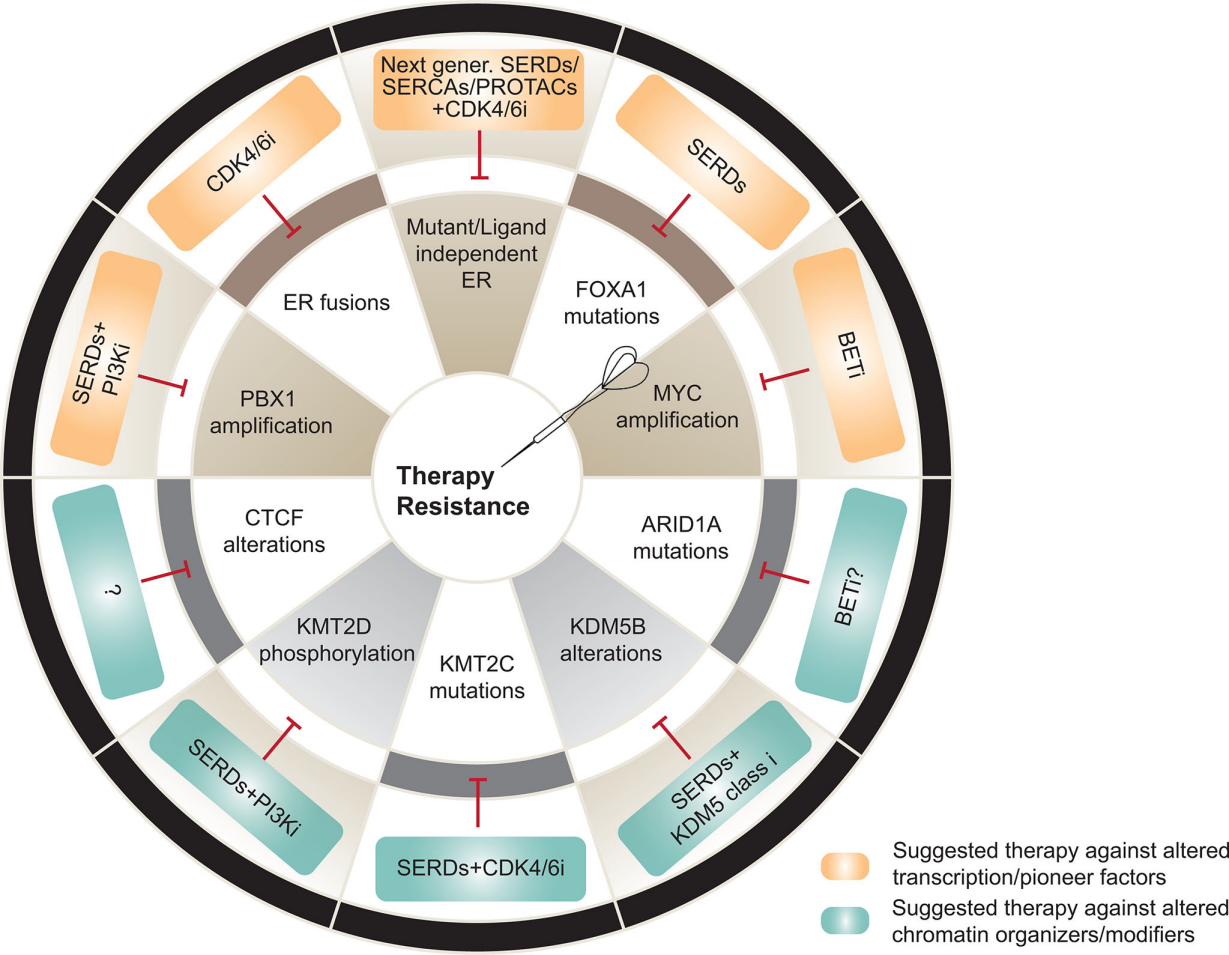
Transcriptional and epigenetic mechanisms of therapy resistance and potential therapeutic alternatives to overcome them. Top, Alterations in transcription and pioneer factors involved in resistance (brown) and suggested therapeutic strategies against the driven mechanisms (orange). Bottom, Alterations in chromatin organizers and modifiers associated to resistance (grey) and suggested therapeutic strategies against the driven mechanisms (blue). SERD, selective estrogen receptor degraders; SERCA, selective ER covalent antagonists; PROTAC, proteolysis-targeting chimeras. Figure adapted and modified from (5).

Our work and others have also shown that genomic disturbances in *FOXA1* can alter ER transcriptional dynamics, driving endocrine therapy resistance. Specifically, activating missense mutations in the Wing2 loop (e.g. H247Y, S250F, F266L) increase the recruitment of FOXA1 to ER cis-regulatory elements and enhance ER-mediated transcription. Breast cancer-specific mutation SY242CS, on the other hand, incites chromatin accessibility changes, leading to the induction of alternative transcriptomes. Moreover, these and other hotspot mutations were found to be mutually exclusive with *ESR1* mutations, and associated with a poorer response to AI therapy in patients (44). In the same line, gene amplification or mutations at the *FOXA1* promoter induce enhanced FOXA1 expression and resistance to standard of care ER degraders and modulators respectively (45, 46) (**Figure 2**). While recent studies characterized the functional outcome of distinct *FOXA1* alterations *in vitro* in other hormone-related cancers, such as prostate cancer (PCa), further *in vivo* studies are required to assess their effect in response to androgen deprivation therapy (47, 48).

Alterations in a variety of ER-related pioneer factors have also been associated with endocrine therapy resistance. One example is GRHL2, a transcription factor classically known for its involvement in epithelial morphogenesis and differentiation, that has been recently characterized as a pioneer factor (49). This novel chromatin opener is enriched at ER loci and cooperates with FOXA1 to drive endocrine therapy resistance in luminal breast cancer. Moreover, increased GRHL2 protein levels are associated with reduced responsiveness to tamoxifen treatment (50, 51). This transcription factor is also amplified in prostate cancer, where it colocalizes with and regulates AR. Its role in the therapeutic response in this context however is ill-defined (52). Another important example is PBX1, which co-occupies 85% of ER loci. Magnani et al. showed that, in fact, this transcription factor is necessary to induce an estrogen-dependent transcriptome distinct from that activated by FOXA1. In line with this, FAIRE-seq experiments revealed that PBX1-bound chromatin is rendered accessible in the absence of estrogen stimulation, demonstrating its pioneering capacity (53). PBX1 has also been shown to regulate ER-dependent transcription upon PI3K inhibition and to sensitize breast cancer cells further to alpelisib (43). Moreover, PBX1 is known to be amplified in metastatic ER+ breast cancers. Importantly, disease-free survival analysis of luminal breast cancer patients from TCGA uncovered PBX1 amplification as a potential biomarker with prognostic value, while the family member with prognostic capacity in PCa has been suggested to be PBX3 (54, 55). PBX1 mediates the expression of a unique NOTCH3 transcriptome that drives endocrine therapy resistance and reduces metastasis-free survival in ER+ cancers (56).

Another bona fide pioneer factor for ER is GATA3, which is mutated in 17% of ER+ metastatic breast cancers (5). However, whether these genomic alterations predict better or worse prognosis in breast cancer remains a controversial issue. In fact, while some studies suggest that *GATA3* mutant tumors might have better overall survival (OS) (57), other groups found no difference in OS, but observed better prognosis for *GATA3* WT tumors (58). Moreover, a comprehensive massively parallel sequencing analysis of 77 tumors suggested that *GATA3* mutations could be positive predictive markers for aromatase inhibitor response (59). The limited experimental evidence suggests that frameshift alterations in this gene might provide a growth advantage compared to cells harboring the WT version. However, these experiments were only conducted in the context of estrogen supplementation, and had no effect on sensitivity to a panel of endocrine treatments or chemotherapies. Furthermore, the mutational repertoire represented in this study only covered a specific frameshift mutation in the ZF2 domain, while leaving most of the truncating alterations in *GATA3* unexplored (31, 60). Evidence suggests that GATA2 is the family member with an equivalent role in regulating the AR cistrome in prostate cancer (61). In addition, GATA2 expression is upregulated upon chemotherapy, driving CRPC aggressiveness (62).

ER activity is also regulated by the pioneer function of AP-2. The gene encoding for this transcription factor, TFAP2, is amplified in 4% of luminal breast cancers and its gene and protein expression levels are associated with worse progression free survival (PFS) upon fulvestrant treatment (63). Magnani and colleagues identified another TF, namely YY1 to be associated with clonal enhancers and promoters in breast cancer patients and as a novel critical determinant of ER transcriptional activity (64).

The pro-oncogene MYC is frequently amplified and is a driver of aberrant proliferation and aggressiveness in many tumor types, including basal breast cancers (65). Along with its well-characterized role in triple-negative or basal-like cancers MYC has also been associated with endocrine therapy resistance mechanisms (66, 67). Another important regulator of the ER transcriptional program is the DNA-binding protein CTCF, which is also found to be mutated in luminal breast cancers (20, 68). Recently, single-nucleotide variation (SNVs) at CTCF binding sites have been associated with altered interaction patterns and transcription of ER target genes, leading to endocrine therapy resistance (69). Resistance-associated SNVs were also strongly enriched at ER binding sites. ER reprogramming in endocrine resistant cells was associated with rewiring of ER-bound interactions between active enhancers and promoters and aberrant expression of these target genes, with many of them being involved in ER-signaling and therapy outcome (69). This work suggested that 3D epigenome remodeling may be an important mechanism underlying endocrine therapy resistance in ER+ breast cancer.

Treatment with PI3K (43) or CDK4/6 inhibitors (70) have also been shown to remodel the chromatin landscape of breast cancer, specifically at enhancers. In regards to CDK4/6 inhibitors, AP-1 transcription factors were upregulated on treatment, which in turn were implicated with widespread enhancer activation in breast tumor models (70). PI3K inhibitors on the other hand enhanced chromatin accessibility at ER cis-regulatory elements (discussed at section 6) (43). Further work is needed to delineate the chromatin landscape of breast tumors resistant to PI3K or CDK4/6 inhibitors.

Finally, several coregulators involved in the activation or repression of the ER machinery have been reported to be recurrently altered in metastatic breast cancers. Examples are NCOR1 (71), RUNX, RARA and AP1. However, functional evidence has yet to be gathered in order to establish them as drivers of endocrine therapy resistance. The case is similar for other transcription factors reported to be mutated in metastatic breast cancers, such as TBX3 or CBFB (31, 33).

For a recent comprehensive review on endocrine therapy resistance mechanisms see Hanker and colleagues (5).

### Alterations in Chromatin Remodelers Impacting Endocrine Therapy Outcome

Truncating mutations in *ARID1A* imply loss of function of this SWI/SNF chromatin remodeling complex subunit in ER+ breast cancers (31, 32). Loss of function mutations or deletions in the SWI/SNF nucleosome remodeling components ARID1A and ARID2 are also enriched in metastatic endocrine-resistant breast cancer (31). We and others recently reported that ARID1A loss is associated with a shorter response to SERDs (11, 42). Mechanistically, ARID1A loss reduces chromatin accessibility and SWI/SNF complex binding at the loci of luminal-determining TFs like FOXA1, ER, and GATA3, resulting in a downregulation of luminal gene signatures and a subset of estrogen regulated genes. These findings may provide an explanation for the longstanding clinical observation that ER+ breast tumors exposed to therapy eventually lose ER and become endocrine therapy resistant. Therapeutic pressure may enable the emergence of cells harboring loss of function mutations in *ARID1A* that confer independence from ER (**Figure 2**). Enhancer reprogramming which promotes phenotypic plasticity and endocrine therapy resistance in breast cancer has also been observed to be mediated by the coordinated role of GATA3 and AP1 TFs which re-organize enhancer landscape promoting tumor phenotypic plasticity (72). Prostate cancer also utilizes similar mechanisms to overcome androgen- and AR target therapies. It has been shown that lineage plasticity can also promote anti-androgen resistance through the SOX2 transcription factor in a TP53-and RB1 loss background in prostate cancer (73). We anticipate that additional alterations in epigenetic and transcriptional regulators are responsible for lineage plasticity upon therapy in hormone-driven cancers.

Sensitivity to endocrine therapies is impacted in a similar fashion by the perturbed action of chromatin modifiers, such as histone methyltransferases or demethylases. For instance, loss of KMT2C (namely MLL3), one of the six members of the SET family of histone lysine methyltransferases, is reported to drive hormone independence in ER+ breast cancer. KMT2C is one of most mutated or deleted genes in ER+ breast cancer patients, and is associated with shorter disease-free survival upon estrogen deprivation with AIs. Despite the advantage of KMT2C-depleted cells in estrogen-deprived conditions, these cells remain ER-dependent and thus, sensitive to therapies involving ER degraders or modulators (74). Another member of the family, KMT2D, happens to be frequently mutated in this cancer type. While there is not enough scientific evidence to relate these alterations to endocrine therapy sensitivity, our group demonstrated that loss of KMT2D sensitizes breast cancer further to PI3K inhibitors through the downregulation of the ER signaling cascade (43). Thus, it is tempting to hypothesize that loss of function mutations in KMT2D might increase sensitivity to ER-targeted therapies. On the other hand, H3K4 demethylases, such as KDM5 (or JARID1B), have been established as oncogenes in luminal ER breast cancer due to their frequent amplification or overexpression (75). In fact, high levels of KDM5 are reported to increase transcriptional heterogeneity, leading to selection of pre-existing resistant clones and poor prognosis in ER+ breast cancers (76).

## Cross-Talk Between Signaling Pathways and Hormone Receptors

One of the first evidences of PI3K and ER signaling crosstalk came from the Breast Cancer Trials of Oral Everolimus-2 (BOLERO-2) phase III clinical trial which demonstrated improvement in progression-free survival (PFS) in endocrine resistant ER+ breast cancer patients treated with the mTOR inhibitor everolimus and exemestane (24). As the first PI3K inhibitors were emerging in the clinic, we and others studied their effects on ER signaling with the goal of identifying the most effective combinatorial therapy for ER+/*PIK3CA* mutant breast cancer. In this regard, we observed a highly uniform adaptive mechanism, orchestrated by the activation of ER signaling upon PI3Kα inhibition, that limited sensitivity to PI3K inhibitors and could be reversed by the addition of endocrine therapy (77). These preclinical findings paved the way for phase III clinical studies testing the PI3Kα inhibitor alpelisib with fulvestrant in patients with metastatic *PIK3CA*-mutant ER+ breast cancer and culminated in the approval of alpelisib by the Food and Drug Administration (FDA) in 2019 (4). Of note, in prostate cancer, which is also dependent on AR and PI3K signaling, it has been shown that inhibition of the PI3K pathway activates AR signaling to support tumor survival. Thus, inhibition of oncogenic PI3K increases tumor growth by unleashing ER/AR signaling in breast and prostate respectively. Mechanistically, PI3Kα inhibition enhances ER signaling through loss of phosphorylation of the epigenetic regulator KMT2D by the PI3K effectors AKT and SGK (43, 78), providing a rationale for epigenetic therapy in combination with PI3K inhibition in this setting. For recent reviews on PI3K inhibitors for cancer therapy see (79–81).

HER2 overexpression has been shown to mediate resistance to endocrine therapies through activation of PI3K or MAPK signaling pathways and thus, ER+ HER2+ patients are currently treated with endocrine therapy in combination with HER2 inhibitors (5). More recently, HER2 activating mutations were found in ~5% of endocrine-resistant metastatic breast cancer (31) and were shown to play active roles in driving resistance (29). Combining anti-HER2 therapy neratinib with fulvestrant has proved to be an effective therapeutic strategy for these tumors (30). In addition, alterations in the MAPK pathway such as NF1 loss are frequent in endocrine resistant ER+ metastatic breast cancer (31), and contribute to resistance to fulvestrant *via* both ER-dependent and ER-independent mechanisms (82). Moreover, nuclear RTKs like FGFR1 have also been shown to influence gene expression in ER+ breast cancer and mediate endocrine therapy resistance (83).

## Collaborative Crosstalk of Nuclear Hormone Receptors

Nuclear receptors events in breast cancer have been generally studied as single receptor chromatin binding events. However, it has become apparent that nuclear receptors collaborate with each other to influence each other binding and therapeutic response with greater complexity than previously recognized. Carroll and colleagues (84) have reported that activated progesterone receptor (PR) can reprogram ER enhancer landscape and that progesterone inhibits estrogen-mediated growth of ER increasing the anti-proliferative effects of endocrine therapy. AR has also been shown to facilitate ER chromatin binding and AR inhibition reduced estradiol-mediated proliferation in ER+/AR+ breast cancer cell lines and synergized with tamoxifen and fulvestrant (85). The role of glucocorticoid receptor (GR) and its post-translational modification of GR such as SUMOylation has been also shown to induce or repress a number of ER binding events and potentially influence decisions on breast cancer therapies (86). However, it is still unclear the chromatin-based mechanisms associated with these crosstalk among nuclear receptors.

## New Avenues of Epigenetic Therapy

Precision oncology efforts have led to the development of epigenetic drugs and nine drugs are FDA-approved including inhibition of EZH2, IDH, DNMTs, and HDACs. Multiple others are in clinical trials for both solids and hematological malignancies. In ER+ breast cancer, phase II trials (NCT00676663, NCT04190056, NCT00828854) are testing therapeutic efficacy of epigenetic drugs with standard of care therapies. Recently, the HDAC inhibitor entinostat has been explored to re-sensitize ER+ tumors to endocrine therapy (ENCORE301) (NCT00676663) but unfortunately has failed to overcome resistance (results presented by M Connolly et al, San Antonio Breast Cancer Symposium, 2020). HDAC inhibitors are also in clinical trials in combinations with CDK4/CDK6 inhibitors (ribociclib, NCT04315233) in triple negative breast cancer. Histone acetylation catalyzed by histone acetyltransferases such as p300/CBP have been shown to be increased in endocrine resistant breast cancer cells highlighting a need to better understand the role of protein acetylation in breast cancer (7). Interestingly, selective inhibitors against p300/CBP, namely CCS1477 has been shown to inhibit the AR transcription program and is currently being evaluated in clinical trials for metastatic castration resistance prostate cancer (NCT03568656). A better understanding of the epigenetic mechanisms influencing breast cancer progression and therapeutic response will be needed for novel drug discovery efforts and rationale-combinatory treatments.

We have learned thus far, that epigenetic regulators have been implicated in endocrine therapy resistant tumors where they can affect ER-dependent transcription, alter the network of ER cofactors or its crosstalk with other signaling pathways, or induce lineage differentiation to promote tumorigenesis. For instance, tumors with high KDM5B have been associated with a shorter response to endocrine treatment, suggesting that inhibitors of the KDM5 family could improve the response to endocrine agents. Likewise, loss of function mutations in KMT2C or mutations in *FOXA1* have been associated with a shorter response to AIs, making ER degraders such as fulvestrant the optimal therapeutic option for the tumors harboring these alterations. In the case of *ESR1* LBD mutations, decreased response to AIs is accompanied of a reduced sensitivity to fulvestrant (35) requiring alternative strategies, such as next generation SERDs, (SERCAs) or PROTACs (19, 87–90).

Other studies have shown how loss of function mutations in *ARID1A* are associated with SERD resistance (42, 91). One of the therapeutic strategies explored in *ARID1A* mutant cancers has been synthetic lethality. To this end, Carroll and colleagues have suggested exploiting synthetic lethality-based treatment strategies in *ARID1A* mutant cancers using inhibitors of BET proteins (91). Similar strategies have been proposed for *ARID1A* mutant ovarian cancer targeting the methyltransferase EZH2 (92). Epigenetic regulators such as KMT2D have also been shown to sensitize ER-driven tumors further to PI3K inhibitors suggesting that small molecule inhibitors against KMT2D could be a promising therapeutic choice in combination with PI3K inhibitors and endocrine therapy (43). Indeed, the development of small molecules that target chromatin regulators has emerged as an active area of current drug discovery efforts. For instance, given the *KAT6A* amplification in 10-15% of breast cancers, novel compounds against KAT6A/KAT68 (PF-9363) have been developed and analyses in preclinical models demonstrate potent anti-tumor activity in ER+ breast cancer cells and xenografts with KAT6A dysregulation (93).

## Conclusion

Over the past decade, the field of transcription and chromatin regulation has grown tremendously and new chromatin-associated processes have emerged as drivers of tumor development and therapeutic response in hormone-driven cancers. These findings have been potentiated by the genomic, transcriptomic, whole-exome, and chromatin accessibility sequencing of breast tumors and preclinical mechanism-based studies using CRISPR-Cas9 screens and whole-genome epigenomic sequencing such as HI-C, CUT & RUN, ATAC-seq and others. Specifically, genomic and transcriptomic analyses of primary breast cancer tumors and matched metastases, coupled with highly curated clinical data, from MSK-IMPACT or AURORA (BIG) initiatives have identified alterations in epigenetic regulators enriched in relapsed metastatic breast cancer (32, 94, 95). A number of these chromatin regulatory processes have begun to be validated and mechanistically delineated in the lab. The systematic integration of such multi-omics analyses of paired biopsies in clinical practice coupled with preclinical mechanistic validation will allow the identification of uncharacterized epigenetic drivers of breast tumorigenesis and therapeutic outcome. In addition, the rapid adoption of technologies that detect circulating tumor-derived cfDNA along with single-cell RNA/ATAC-sequencing will be important to capture the molecular heterogeneity of treatment resistance. Given that some of the genomic mechanisms of endocrine resistance have been found to be at low frequency, future efforts will require greater patient sample size and a focus not only on genomics on a panel of genes but whole-exome sequencing and transcriptomics and chromatin accessibility analyses to provide signatures of therapeutic resistance and response. These efforts would be facilitated by multi-institutional and cooperative data sharing efforts similar to the AURORA initiative. Finally, the identification of novel epigenetic regulators as drivers of breast tumorigenesis and therapeutic response will allow the rational design of novel inhibitors to overcome resistance. In order for these mechanisms to be suitable targets for cancer therapy, future work will need to identify: i) the tumor subtypes that are highly addicted to the chromatin-based mechanism, ii) rationale-based combinatorial strategies, and iii) optimal dosing and scheduling to increase efficacy and safety. This new and exciting body of evidence together with the systematic and integrative pursuit of multi-omics approaches in preclinical and clinical samples will greatly impact the study of chromatin regulatory systems in breast cancer and the identification of new treatment strategies.

## Author Contributions

AA-A and ET jointly participated in the planning, writing, and editing of the review. All authors contributed to the article and approved the submitted version.

## Funding

AA-A is supported by a Juan de la Cierva Incorporacion fellowship from the Ministerio de Ciencia e Innovacion (Spain) and by a European Research Council Consolidator grant. ET is supported by grants from the JKTG foundation, Breast Cancer Research Alliance, Cancer Research Informatics, and NCI NIH grants K22CA245487 and R21CA252530.

## Conflict of Interest

ET has received honoraria, consulting fees, and a grant from AstraZeneca.

The remaining author declares that the research was conducted in the absence of any commercial or financial relationships that could be construed as a potential conflict of interest.

## Publisher’s Note

All claims expressed in this article are solely those of the authors and do not necessarily represent those of their affiliated organizations, or those of the publisher, the editors and the reviewers. Any product that may be evaluated in this article, or claim that may be made by its manufacturer, is not guaranteed or endorsed by the publisher.
